# Prioritizing social vulnerability in urban heat mitigation

**DOI:** 10.1093/pnasnexus/pgae360

**Published:** 2024-08-30

**Authors:** Kwun Yip Fung, Zong-Liang Yang, Alberto Martilli, E Scott Krayenhoff, Dev Niyogi

**Affiliations:** Department of Earth and Planetary Sciences, Jackson School of Geosciences, The University of Texas at Austin, Austin, TX 78712, USA; Department of Earth and Planetary Sciences, Jackson School of Geosciences, The University of Texas at Austin, Austin, TX 78712, USA; Atmospheric Modelling Unit, Environmental Department, CIEMAT, 28040 Madrid, Spain; School of Environmental Sciences, University of Guelph, Guelph, ON N1G 2W1, Canada; Department of Earth and Planetary Sciences, Jackson School of Geosciences, The University of Texas at Austin, Austin, TX 78712, USA; Maseeh Department of Civil, Architectural, and Environmental Engineering, Cockrell School of Engineering, The University of Texas at Austin, Austin, TX 78712, USA

**Keywords:** urban overheat mitigation, heat waves, social vulnerability, urban trees, Weather Research and Forecasting model

## Abstract

We utilized city-scale simulations to quantitatively compare the diverse urban overheating mitigation strategies, specifically tied to social vulnerability and their cooling efficacies during heatwaves. We enhanced the Weather Research and Forecasting model to encompass the urban tree effect and calculate the Universal Thermal Climate Index for assessing thermal comfort. Taking Houston, Texas, and United States as an example, the study reveals that equitably mitigating urban overheat is achievable by considering the city's demographic composition and physical structure. The study results show that while urban trees may yield less cooling impact (0.27 K of Universal Thermal Climate Index in daytime) relative to cool roofs (0.30 K), the urban trees strategy can emerge as an effective approach for enhancing community resilience in heat stress-related outcomes. Social vulnerability-based heat mitigation was reviewed as vulnerability-weighted daily cumulative heat stress change. The results underscore: (i) importance of considering the community resilience when evaluating heat mitigation impact and (ii) the need to assess planting spaces for urban trees, rooftop areas, and neighborhood vulnerability when designing community-oriented urban overheating mitigation strategies.

**Significance Statement**

Intense heat stress during urban heatwaves poses a grave threat, particularly to vulnerable communities. Consideration of community resilience in designing mitigation strategies for urban overheating is achievable. This study employs city-scale simulations to assess the cooling impact of urban overheating mitigation strategies, while also considering social vulnerability. The results highlight the impacts from city's layout and demographics on the equitability of mitigation strategies. In response to the escalating challenges posed by climate-intensified heatwaves, this research lays the groundwork for city-specific goals aimed at creating community-oriented and diverse mitigation approaches. By addressing the vulnerable communities, such as the elderly and underprivileged households, this study contributes toward efforts for safeguarding public health and advancing environmental justice for climate extremes.

## Introduction

There is a surge in both the frequency and severity (1) of heatwaves (2, 3). Urbanization compounds the impact of heatwaves through the urban heat island (UHI) effect causing urban overheating (4, 5), which can lead to negative impacts on both human health and overall well-being. The UHI intensifies due to factors such as increased building surface area (6, 7), low-albedo materials absorbing more shortwave radiation (8), lack of vegetation for shading and evaporative cooling (9), and anthropogenic heat sources (10). Coastal populous cities such as Houston, Texas, and United States may experience a more pronounced daytime UHI effect compared with inland US cities, largely due to their sensitivity to sea breeze and the frequency of extreme heat days (11). However, the intensity of UHI effect can also depend on various factors such as city size and urban morphology. In this study, we selected Houston due to the observed strong daytime UHI effect. Research indicates that severe heatwaves in Houston show a marked rise in emergency department visits (12), and projections predict a significant escalation of the daily maximum heat index by 2040, substantially heightening heat exposure for the future urban population (13).

Amid heatwaves, some demographics bear disproportionate consequences. Vulnerable groups such as the elderly and those with chronic illnesses face elevated mortality risks (14, 15). Studies emphasize that poverty and subpar housing quality can amplify heat-related mortality rates (16, 17) largely due to disproportionate exposure in low-income neighborhoods marked by limited greening spaces (18–20). Consequently, the need for community-oriented mitigation strategies to tackle urban overheating becomes imperative.

To develop community-oriented strategies, it is crucial to understand the vulnerability of a city. Vulnerability consists of both sensitivity and adaptive capacity. Sensitivity relates to the susceptibility of a community toward hazards, and adaptive capacity relates to the resilience of a community toward hazards. The Social Vulnerability Index (SVI (21)) from the US Centers for Disease Control and Prevention can be used to quantify the vulnerability of US cities down to the scale of census tracts. The SVI quantifies census tracts’ vulnerability based on sensitivity factors (e.g. socioeconomic status, household composition, and minority status) and adaptive capacity factors (e.g. housing type and transportation). The SVI has a range from 0 to 1 with higher values indicating higher vulnerability. For better communication, in this study, we will use the word “neighborhoods” to represent the area cover by census tracts. Research confirms that high SVI neighborhoods experience more emergency medical service requests during heatwaves (22).

To tackle urban overheating, a range of mitigation approaches are available. Cool roofs, green roofs, and urban trees are some prominent strategies. Cool roofs reflect sunlight, minimizing heat absorption and lowering near-surface temperatures (23–25). Green roofs utilize rooftop vegetation to enhance evapotranspiration, reducing daytime temperatures (26–28). Urban trees, planted along streets, provide cooling through shading and evapotranspiration, contributing to the reduction in near-surface temperatures (29–32). The cooling magnitudes of these strategies are generally dependent on the setup such the albedo changes and amount of green coverage.

Various indices have been developed to measure heat stress and human thermal comfort (33). Operational indices such as the heat index (34) and wind chill temperature (35) offer insight into how humans experience comfort in extremely hot and cold conditions, respectively. However, these indices overlook the influence of radiation. This study addresses this gap by employing the Universal Thermal Climate Index (UTCI (36)), which derives its thermal assessment from the human energy balance equation, by incorporating factors such as temperature, relative humidity, wind speed, and radiation components. Notably, unlike operational indices, the UTCI aptly captures the human body's sensitivity to ambient variations and remains adaptable across diverse weather conditions (37, 38).

**Fig. 1.**
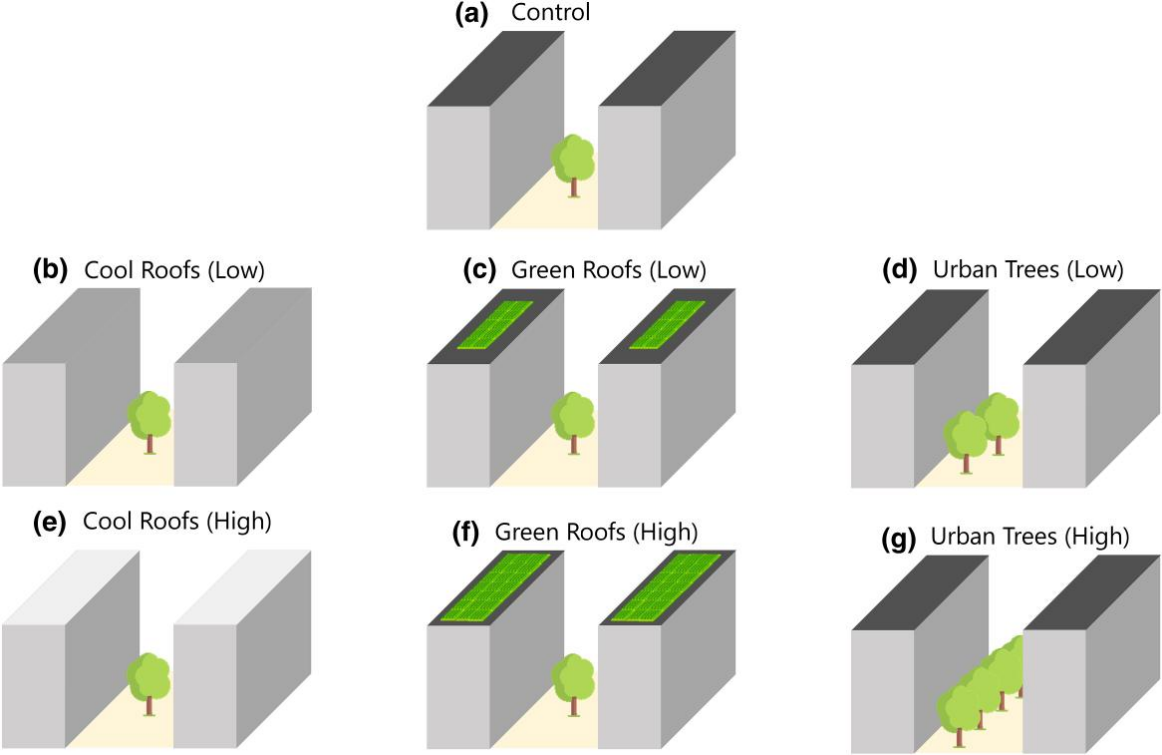
Schematic diagram for simulation cases. a) Control: roof albedo ≈ 0.19, green roof coverage = 0%, and street tc ≈ 17%, mirroring real-world conditions. b) Cool roofs (low): green roof coverage and street tc remain unchanged from control, while roof albedo is moderately raised to 0.55. c) Green roofs (low): roof albedo and street tc retain control values, with green roof coverage slightly elevated to 30% of the roof area. d) Urban trees (low): roof albedo and street green roof coverage are consistent with control, but street tc experiences a minor increase to ≈ 24% of the street area. e) Cool roofs (High): green roof coverage and street tc remain unchanged from control, while roof albedo sees a notable increment to 0.70. f) Green roofs (high): roof albedo and street tc remain consistent with control, with a substantial increase in green roof coverage to 80%. g) Urban trees (high): roof albedo and street green roof coverage remain unchanged from control, while street tc sees a moderate elevation to ≈ 31%.

Within this study, we enhanced the Weather Research and Forecasting (WRF (39)) model with modifications to encompass the urban tree effect. By simulating five distinct heatwave incidents in Houston, we assessed the combined outcomes of these events (see “Methods” section regarding the methodology for identifying the heatwave events and Table S1 for the summary of the simulated events). Our analysis centered on the evaluation of shifts in thermal comfort using the UTCI. We compared the performance of cool roofs, green roofs, and urban trees mitigation strategies against control (CTL) experiment (see “Methods” section and Table S2 for the model setup). Our overarching objectives were to: (i) gauge the efficacy of these mitigation approaches in thermal comfort during heatwave occurrences and (ii) simultaneously examine their implications for promoting community resilience by addressing social vulnerability. In this study, we assessed the effectiveness of three strategies to mitigate overheating: (i) cool roofs, (ii) green roofs, and (iii) urban trees. For each mitigation strategy, we implemented both a high-scenario experiment, which involved higher albedo, green roof coverage, or tree coverage (tc), and a low-scenario experiment, characterized by lower albedo, green roof coverage, or tc. Altogether, one control and six representative urban overheating mitigation strategy experiments were conducted (see “Methods,” Fig. 1, and Table S3 for the details of experimental setup).

## Results

### Cool roofs create most significant daytime cooling

**Fig. 2.**
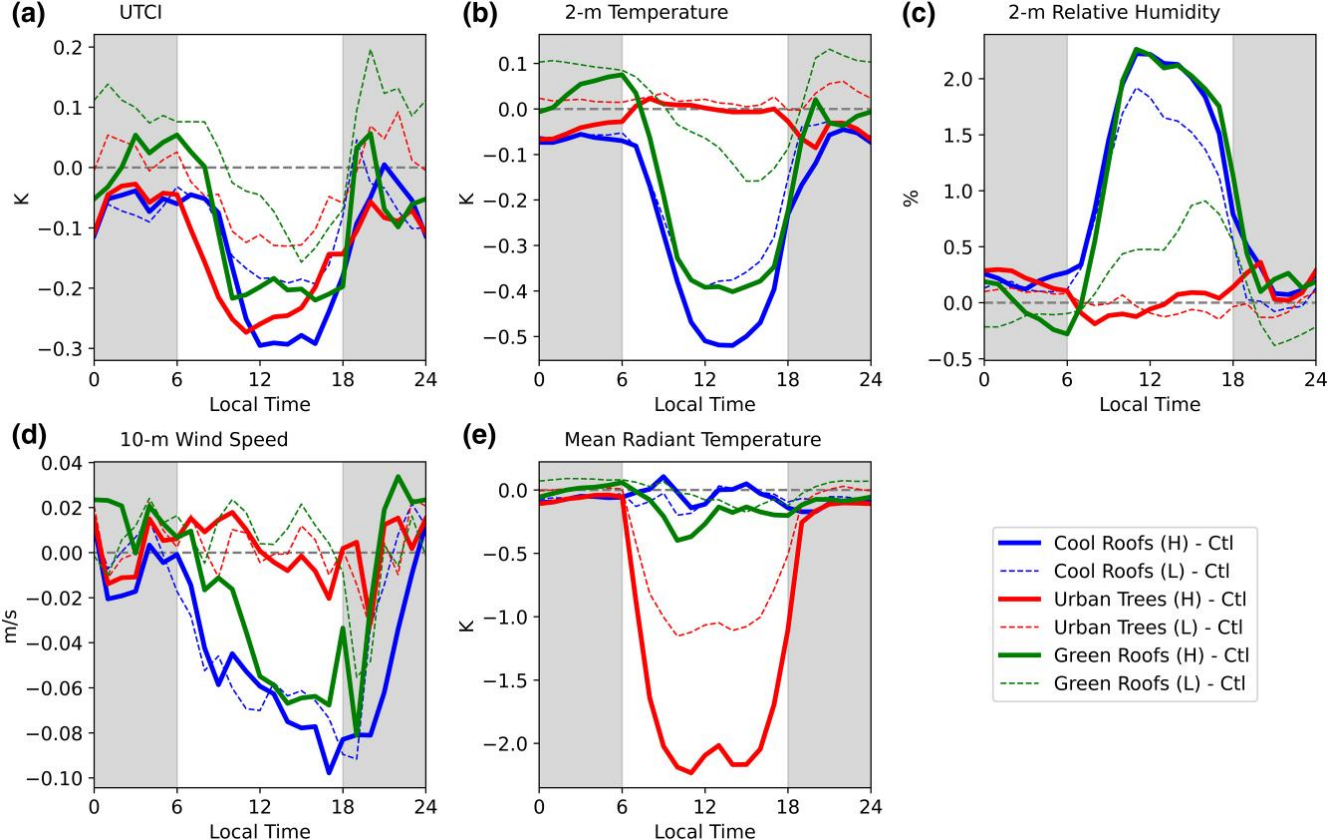
Diurnal variations in thermal comfort index and meteorological variables from urban overheating mitigation strategies. a) UTCI. b) 2-m temperature. c) 2-m relative humidity. d) 10-m wind speed. e) Mean radiant temperature. These variables are calculated as the average over the urban grids and amalgamated from five simulated heatwave events.

We evaluated the changes in thermal comfort (UTCI) across the urban overheating mitigation strategy experiments as compared to control experiments, averaged over the entire urban expanse in Houston. The UTCI calculations (Fig. 2a) encompass four integral components: temperature (Fig. 2b), relative humidity (Fig. 2c), wind speed (Fig. 2d), and mean radiant temperature (Fig. 2e). Notably, a reduction in temperature, relative humidity, mean radiant temperature, coupled with an increment in wind speed, leads to a reduction in UTCI, instigating a cooling influence.

Our initial WRF simulations underscore that high-scenario implementations consistently yield more robust daytime cooling effects in contrast to low-scenario counterparts. To facilitate a more insightful comparison of mitigation strategies, we exclusively present outcomes from the high-scenario simulations later. It emerges that cool roofs yield the largest daytime cooling (0.30 K), followed by urban trees (0.27 K) and then green roofs (0.22 K; Fig. 2a). Although each strategy provides cooling benefits, their underlying mechanisms diverge. Cool roof and green roof strategies reduce the UTCI by mitigating temperature (Fig. 2b), despite increased relative humidity (Fig. 2c) and diminished wind speed (Fig. 2d). Urban trees, however, exhibit a distinctive cooling mechanism driven by the reduction in mean radiant temperature (Fig. 2e). This is a result of evapotranspiration-induced cooling and tree shading that blocks substantial solar radiation, with no significant deviations in temperature, relative humidity, and wind speed. Importantly, our findings underscore that the traditional temperature and heat index metrics, encompassing temperature and relative humidity, inadequately capture the cooling effect stemming from urban tree evapotranspiration and shading.

### Urban trees as the optimal strategy to address social vulnerability in mitigating heat stress

**Fig. 3.**
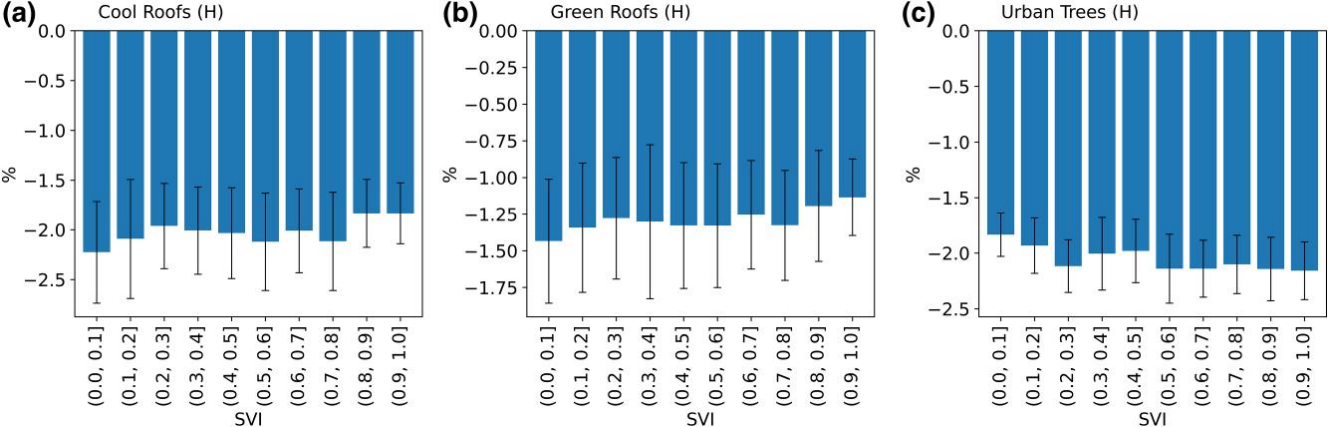
The percentage changes in ADCH aligned with vulnerability levels for high-scenario strategies. a) Cool roof strategy outcomes. b) Green roof strategy outcomes. c) Urban tree strategy outcomes. The error bars in the graphs denote the spread of the interquartile range, providing insights into the variability of the data points.

To evaluate the equitability of the mitigation strategies in alleviating heat stress, we evaluated the averaged daily cumulative heat stress (ADCH; see “Methods” for detail calculations) alterations attributed to these strategies relative to neighborhoods’ SVI. Our findings reveal an important cautionary pattern: both cool roof and green roof strategies show that the effectiveness in the ADCH decreases as the SVI increases, indicating a diminish improvement in thermal comfort for more vulnerable neighborhoods (Fig. 3a and b). On the other hand, the urban trees strategy demonstrates a more pronounced improvement in thermal comfort (decreased ADCH) with increasing SVI (Fig. 3c), from 1.7% reduction in ADCH in the resilient neighborhoods (census tracts with SVI < 0.1) to 2.2% reduction in the vulnerable neighborhoods (census tracts with SVI > 0.9). This suggests that green cover has higher potential in alleviating heat stress equitably. To highlight the significance of a 2% reduction in ADCH, this might encompass either a temperature-related cooling effect or a shortened duration during heatwave events. For instance, considering a UTCI threshold of 26°C to denote heat stress, a 2% reduction could signify cooling for 24 h at 40 to 39.7°C, or a duration reduction of 28.8 min while maintaining a UTCI of 40°C.

As an innovative endeavor to rank equitability among strategies, we introduce the vulnerability-weighted daily cumulative heat stress change (VDCH). By multiplying cumulative heat stress changes from mitigation strategies with SVI (see “Methods” section for more details), this metric can quantitatively rank the equitability of strategies. VDCH values hold meaningful interpretations: VDCH = 0 denotes the negligible impacts on community heat stress resilience, owing to either minimal heat stress changes or low neighborhood vulnerability. VDCH > 0 and VDCH < 0 indicate the deteriorated and improved community heat stress resilience, respectively. Our outcomes highlight urban trees as the optimal strategy (−1.41%), followed by cool roofs (−1.28%) and green roofs (−0.67%), for fostering community resilience and explicit heat stress mitigation during daytime (Table 1).

**Table 1.** The percentage changes in urban-averaged VDCH during the whole day, daytime, and nighttime for each mitigation strategy.

Experiment	Whole day (%)	Daytime (%)	Nighttime (%)
Urban trees (high)	−1.06	−1.41	−0.87
Cool roofs (high)	−1.03	−1.28	−0.87
Green roofs (high)	−0.65	−0.67	−0.68

### City's planting spaces, roof areas, and vulnerability are the key to design community-oriented mitigation strategies

**Fig. 4.**
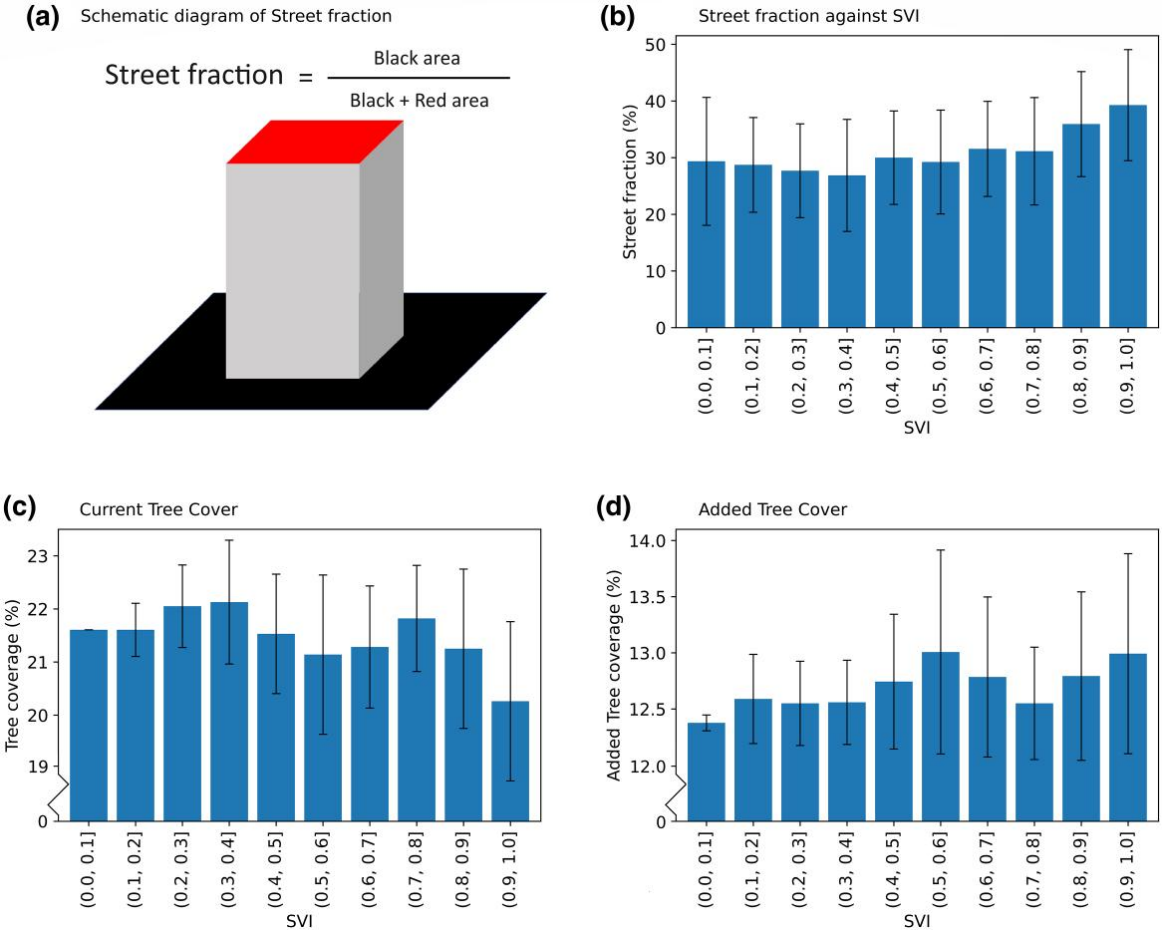
Unveiling the factors driving community-oriented urban overheat mitigation strategies. a) Schematic diagram of street fraction. b) Street fraction versus SVI in urban Houston. c) CTL experiment's tc, derived from observations. d) Added tc from urban trees high-scenario compared to CTL experiment. The error bars encapsulate the spread of the interquartile range.

The robust performance of the urban trees strategy in vulnerable neighborhoods owes itself to dual factors. Firstly, Houston's vulnerable neighborhoods (census tracts with SVI > 0.9) possess greater street fraction (Fig. 4a; derived from the University of Texas-GLObal Building heights for Urban Studies dataset (40)) with about 40%, compared to resilient neighborhoods (census tracts with SVI < 0.1) with < 30% (Fig. 4b). Even when having identical tc addition across neighborhoods, the abundance of planting spaces enhances tree numbers in vulnerable neighborhoods. In computational modeling, an urban grid (summation of black and red area in Fig. 4a) is composed of both the roof area (red area in Fig. 4a) and the street area (black area in Fig. 4a). Therefore, the roof area is inversely correlated with street planting spaces. This, in turn, impairs heat stress reduction from rooftop strategies such as cool roofs and green roofs within vulnerable neighborhoods.

Additionally, our experimentation implemented varying tc based on land-use classes via Tree Equity Score (TES) estimation (see “Methods” for details). This was done by introducing higher tc in areas with greater vulnerability (areas with higher SVI) under the urban tree strategy. Our findings indicate that the CTL experiment, reflecting the current reality situation, has less tc in the areas with greater vulnerability (Fig. 4c). In contrast, the urban tree strategy introduces additional tc within these areas (Fig. 4d). Hence, the urban tree strategy can create a stronger cooling in the areas with greater vulnerability. Our results show that the strategy exhibiting the most robust cooling effect (cool roofs) might not necessarily be the optimal choice for alleviating heat stress (urban trees) for some socially vulnerable communities. Central to our findings is the crucial role played by urban morphology and demographics in designing community-oriented solutions. Specifically, understanding changes in street fraction, which determines tree planting space availability and roof spaces for rooftop strategies, in relation to neighborhood vulnerability is important.

## Discussion

The urban tree module embedded in our model, while insightful, harbors simplifications and inherent constraints. Notably, the model only accounts for converting intercepted energy from tree leaves into either sensible heat or latent heat, omitting the conversion into longwave radiation (41). This might potentially impact the tree's cooling effect. Additionally, the model overlooks the diminished wind speed caused by tree drag, potentially resulting in an overestimation of trees’ cooling effect. Furthermore, the model does not incorporate trees’ thermal storage capacity, which could underestimate nighttime warming (42), a phenomenon similar to the impact of green roofs. In our study, we also found that green roofs can induce nighttime warming (Fig. 2a and b). This warming, as documented in prior research (23, 27, 28, 43–45), arises from the additional soil layer's elevated heat capacity, which accumulates heat during daylight hours and releases it into the atmosphere during nighttime. Notably, the high-scenario green roof strategy mitigates this nocturnal warming effect, owing to strengthened daytime cooling that reduces energy absorption. Consequently, the magnitude of energy released into the atmosphere during nighttime diminishes (27). Besides the physical processes, the current urban tree module does not consider the differences in characteristics among tree species, such as the leaf area index (LAI) and transpiration sensitivity toward soil moisture, which may affect the cooling effectiveness when different types of trees are planted. However, these limitations do not compromise our study's essence: demonstrating how to evaluate the equitability of urban overheating mitigation strategies.

Even though cooling efficacy hinges on mitigation strategy magnitude: albedo for cool roofs, rooftop vegetation coverage for green roofs, and street tc for urban trees, our findings persistently emphasize the necessity of integrating city demographics and morphology in formulating community-oriented urban overheating mitigation strategies. Urban trees may not invariably represent the epitome of community-oriented strategy. A case in point is Maricopa County, Arizona, where a study indicates uniform cool roof implementation can yield more potent cooling in regions populated by heat-sensitive individuals (e.g. persons of young age, disability, or chronic illnesses) due to higher roof area fractions (46). As depicted in the schematic in Fig. 4a, the inverse relationship between roof area fraction and street fraction implies that, in certain contexts like Maricopa County, rooftop mitigation strategies might indeed surpass urban trees in terms of addressing social vulnerability. However, this discussion does not rule out the cooling potential brought by the urban tree in Maricopa County.

Under climate change, warmer temperatures and changes in precipitation patterns will threaten the survival rate of vegetation, potentially impacting the effectiveness of cooling from the urban trees strategy. Studies show that globally, 76 and 70% of tree species will be at risk in 2050 due to changes in temperature and precipitation, respectively (47). The low-scenario urban trees strategy (50% of additional trees survival rate) in our study shows that if only 50% of the trees were added, the cooling effectiveness is reduced by half (Fig. 2a). Therefore, more investigations have to be carried out to evaluate the sensitivity of tree species to climatic changes and also explore the effectiveness of joint urban overheating mitigation strategies. Furthermore, from a socioeconomic perspective, the cost of tree maintenance will increase in the future due to more tree species can be at risk. The benefit–cost ratio of urban tree species will change over time.

To illustrate disparities between rooftop and urban tree strategies, we conducted independent simulations for each. However, to optimize strategy equitability, amalgamating mixed methods merits exploration. Although street fraction experiences an upward trend with the SVI (Fig. 4b), a 10% variation remains evident. By analyzing vulnerable neighborhoods’ morphology, specific regions characterized by high roof and street fractions can be identified. This enables tailored implementation of rooftop and urban tree strategies, respectively, to effectively combat urban heat stress.

## Methods

### Heatwave events selection and simulation

In this study, we engaged five integrated surface dataset (ISD (48)) stations around the Houston area, which have more than 30 consecutive years of measurements (denoted as “Climatology Stations” in Fig. S1a) to discern pertinent heatwave events. Drawing from a continuous 30-year dataset spanning 1990 to 2019, derived from 2-m temperature data, we identified heatwave instances. The criterion for selection involved calculating the station-averaged daily maximum temperature during the summer months (June–July–August). Hence, a threshold of 32.1°C was established. Days surpassing this temperature were categorized as hot days and further sorted into heat episodes if consecutive. To identify the prolonged heatwave events, episodes extending beyond 5 days were singled out as heatwave cases, which is much longer than the US National Weather Service's definition of 2 days of abnormally hot and humid weather (49) and Burrow's definition of 3 days of maximum temperature exceeding 90°F (50). Focusing on recent years, a total of 12 such cases between 2017 and 2019 were identified using this approach.

From these cases, there are five heatwave events that exhibit daily maximum temperatures surpassing 35.1°C (fulfilling Burrow's definition of heatwave). At the same time, these five events also have the longest durations, which extended beyond 14 days. Therefore, we have selected these five events for simulation. These simulated heatwave events adhere to the World Meteorology Organization's heatwave definition—comprising more than 5 consecutive days of daily maximum temperature exceeding the average daily maximum by 5°C, which Houston has an average daily maximum temperature of 25.6°C.

We identified the peak daily maximum temperature within each simulated heatwave event, typically occurring at 21Z, corresponding to 15:00 Central Standard Time. Simulation coverage extended ∼48 h before and after the peak. Detailed information of these simulated cases is available in Table S1.

### WRF simulation setup for heatwave events

The simulation of the five identified heatwave events employed WRF version 4.4, incorporating the advanced WRF dynamic core (37). The multilayer urban canopy model, coupled with the building effect parameterization and building energy model (BEP–BEM (51, 52)), was integrated into the framework. Initial and boundary conditions were drawn from the ERA5 reanalysis at a spatial grid spacing of 0.25° and a temporal interval of 6 h.

Our simulation adopted a three-domain two-way nested structure, each encompassing distinct grid spacings: 9 km (d01; 160 × 163), 3 km (d02; 238 × 235), and 1 km (d03; 331 × 331), with Houston centered in d03. The specific domain arrangement is outlined in Fig. S1b. Vertically, 55 levels were utilized, with the lowest 10 levels having 5-m increments and the uppermost layer terminating at 50 hPa. Model physics parameterizations rooted in prior Houston-based numerical studies (53). The array of parameterization schemes utilized is given in Table S2. To accurately capture synoptic conditions from ERA5, spectral nudging of zonal and meridional wind above 500 hPa was applied within the outermost domain, specifically for wavelengths surpassing 1,000 km (54).

To effectively characterize land categories, we integrated the Houston Local Climate Zone (LCZ) map at 100 m resolution and MODIS MCD12Q1 land-use and land-cover map from 2018 at 500 m resolution (Fig. S1a). The LCZ map was formulated based on five Landsat 8 imageries acquired during 2018 (April 23, May 21, June 19, August 1, and October 18) employing the random forest algorithm. Both the LCZ and MODIS land-use–land-cover maps were regrided into 1 km to match the simulation resolution in d03.

### Enhancing BEP–BEM with street trees consideration

The existing BEP–BEM currently has the capability to integrate cool and green roofs, but urban street trees module is not incorporated yet. While a standalone BEP-Tree model has been developed (55, 56), its integration into the WRF model is pending. To bridge this gap, we have undertaken partial modifications to the radiation aspect, aimed at evaluating the ramifications of street tree shading by considering the standard physics for solar radiation interception by tree foliage (employing Beer's law) and augmented latent heat due to transpiration (57). This approach has previously been published in Stone et al. (57–59). The primary additional assumption here relates to how intercepted solar radiation is directed after absorption. Previously, it was assumed to fully drive transpiration (57–59), a somewhat crude assumption. Here, we apply a user-defined Bowen radiation (based on the climate of Houston) to split this energy between latent (transpiration) and sensible heat fluxes, which provides a more realistic (less idealized) outcome.

An evaluation of the BEP-Tree carried out in London, ON, Canada, and Salt Lake City, UT, USA, also shows that the reduction in downwelling shortwave radiation under the tree canopy during daytime, as measured by the radiometer, can be reproduced by the BEP-Tree model. The sensitivity test of LAI shows that an increase of LAI by 0.8 can reduce downwelling shortwave radiation by ∼ 200 Wm^−2^ and reduce the road surface temperature by ∼ 5 K at the midday (56). We have also carried out a sensitivity analysis for this study by calculating the UTCI changes from urban trees experiment—CTL experiment (see “Experimental setup and parameters estimations” for details). The sensitivity analysis shows that the UTCI is reduced by 0.26°C per 0.1 fraction of tree canopy increase (Fig. S2). Our sensitivity analysis has a similar magnitude of cooling from a meta-analysis showing 0.3°C cooling per 0.1 canopy cover fraction increase (25).

In this adaptation, we address the original shortwave radiation component reaching the ground surface, denoted as $SW_{\mathrm{groun}d_{\mathrm{sun}}}$, as in the absence of trees within the BEP–BEM. To account for the shortwave energy absorbed by street trees, as described in Eq. (1), we update the shortwave radiation reaching the ground surface, $SW_{\mathrm{ground}}$, in Eq. (2):


(1) $$\begin{array}{rl} & \mathrm{SW}\;\mathrm{reaching}\;\mathrm{the}\;\mathrm{ground}\;\mathrm{under}\;\mathrm{the}\;\mathrm{tree}\;\mathrm{cover}:\\ & SW_{{\mathrm{ground}}_{\mathrm{tree}}}=SW_{\mathrm{groun}d_{\mathrm{sun}}}\cdot \exp\;\left(-\frac{\Omega \cdot G\cdot \mathrm{LA}I_{\mathrm{tree}}}{\cos\;(\varphi )}\right). \end{array}$$

(2) $$\begin{array}{rl} & \mathrm{SW}\;\mathrm{reaching}\;\mathrm{the}\;\mathrm{ground}:SW_{\mathrm{ground}}=(1-tc)\cdot \\ & SW_{\mathrm{groun}d_{\mathrm{sun}}}+\mathrm{tc}\cdot SW_{{\mathrm{ground}}_{\mathrm{tree}}}. \end{array}$$

where Ω=0.5 is the empirical clumping coefficient, G=0.5 accounts for the distribution of leaf orientation angles, φ is the solar zenith angle, tc, and $\mathrm{LA}I_{\mathrm{tree}}=3$ is a reasonable LAI for urban street trees. It is important to note that LAI varies among different tree types and across seasons. The Houston vicinity predominately features evergreen broadleaf forest, evergreen needleleaf forest, cropland, and grassland; the forests have an LAI averaging of ∼3 (60).

Following this radiation modification, we assume that all absorbed shortwave energy $(en_{\mathrm{loss}})$ undergoes the conversion into either sensible heat $(SH_{\mathrm{tree}})$ or latent heat $(LH_{\mathrm{tree}})$. Given that energy storage in leaves and woody elements is small, this assumption essentially amounts to assuming that net longwave radiation exchanges between trees and surroundings (sky, ground, and buildings) are close to zero. Here, we apply the Bowen ratio (β) to encapsulate the ratio of sensible heat to latent heat fluxes exchanged by the tree leaves, assuming that it is approximately fixed for the duration of our simulation period:


(3) $$\mathrm{Energy}\;\mathrm{absorbed}\;\mathrm{by}\;\mathrm{trees}:\;en_{\mathrm{loss}}=\;SW_{\mathrm{groun}d_{\mathrm{sun}}}-SW_{\mathrm{ground}}.$$

(4) $$\mathrm{Latent}\;\mathrm{heat}\;\mathrm{release}\;\mathrm{by}\;\mathrm{trees}:\;LH_{\mathrm{tree}}=\frac{en_{\mathrm{loss}}}{\beta +1}.$$

(5) $$\mathrm{Sensible}\;\mathrm{heat}\;\mathrm{release}\;\mathrm{by}\;\mathrm{tree}:\;SH_{\mathrm{tree}}=\frac{\beta \cdot en_{\mathrm{loss}}}{\beta +1}.$$

where β=2.0. In the Houston area, the average tc stands at ∼20% (estimated from the TES (61)), while the corresponding Bowen ratio is ∼2.0 (62, 63).

### UTCI calculation for pedestrian thermal comfort analysis

The UTCI computation relies on sixth-order approximating polynomials encompassing four variables: temperature, relative humidity, wind speed, and mean radiant temperature (36). This study specifically seeks to evaluate shifts in outdoor pedestrian thermal comfort. To this end, the 2-m temperature, 2-m relative humidity, 10-m wind speed, and lowest level mean radiant temperature were utilized to compute UTCI at the street level. Introducing mean radiant temperature as an extra variable added to the WRF model accommodates UTCI changes arising from radiation reaching pedestrians (64).

For each of the two street directions (north–south and east–west), three mean radiant temperatures were calculated in the WRF model based on the radiation entering the urban canyon and the interaction with the urban morphology, representing the sidewalks and center of the street. These values were averaged across all positions. Factoring in subgrid wind speed variation, a distribution of UTCI values was inferred for each grid cell. This distribution facilitated the derivation of UTCI values corresponding to a cool spot (10th percentile), midrange (50th percentile), and hot spot (90th percentile) in the WRF output. Opting for the midrange values enabled the assessment of average conditions. For an in-depth breakdown of the methodology, refer to Martilli et al. (65).

### Experimental setup and parameters estimations

The study encompasses seven distinct experiments, comprising one control and three urban overheating mitigation strategies: (i) cool roofs, (ii) green roofs, and (iii) urban trees. For each urban overheating mitigation strategy, we implemented both a high-scenario experiment, which involved higher albedo, green roof coverage, or tc, and a low-scenario experiment (Fig. 1). Within the CTL experiment, the roof albedo, road albedo, green roof coverage, and tc (Eq. (2)) were derived from observations. Roof and road albedo values were computed from the HLSL30v002 product obtained via the Harmonized Landsat/Sentinel-2 project. For this purpose, data from three clear sky days spanning different seasons in 2017 were considered. Due to the long revisit time for Landsat and Sentinel-2, the cloudless day identified may not match the simulation period. Therefore, the data from March 21, May 24, and September 29 were used to calculate the albedo. Given the close resemblance of visible band wavelengths between Landsat 7 and Landsat 8, the narrowband to broadband albedo algorithm for Landsat 7 was employed to determine albedo (66):


(6) $$\mathrm{Albedo}:\;\mathrm{Albedo}\;=\;0.443\times \mathrm{albed}o_{\mathrm{blue}}+0.317\times \mathrm{albed}o_{\mathrm{green}}+0.240\times \mathrm{albed}o_{\mathrm{red}}.$$

To ascertain overall roof albedo within each LCZ class, the Microsoft building footprint dataset (67) was utilized to identify roof pixels.

Considering the minimal implementation of green roofs in Houston, 0% coverage was assumed in the CTL experiment. Urban tc, gleaned from the 2020 US Tree Map via the TES (61), was census tract-averaged and further averaged by LCZ class. Tree height estimations were derived from the Global Forest Canopy Height 2019 dataset, a product of the Global Ecosystem Dynamics Investigation Lidar measurement (68).

For each of the urban overheating mitigation strategies (cool roofs, green roofs, and urban trees), two scenarios were adopted: low and high intensity. The low scenarios, serving as a mild mitigation or deteriorate scenario, incorporate reduced albedo, green roof coverage, and tc. The lowered albedo in cool roofs signifies scenarios where roofs are coated with diverse colors or dust, diminishing albedo. Reduced green roof and tc correspond to instances where less vegetation is planted or vegetation experiences mortality. Distinctly, the cool roof and green roof strategies involve modifications solely applied to roofs, while the urban trees strategy modifications are confined to the streets.

In the case of the cool roof experiment, the low-intensity scenario was informed by the Houston Cool Roof Guidelines (ASHRAR90.1-2013), assigning a roof albedo value of 0.55. The high-intensity scenario, set to 0.70, followed the Austin Reflective Roofing guidelines (§25-12-263, 502.5). For the green roof experiment, the low-intensity scenario based on the lowest green roof coverage (30%) incentivized by the city of Austin. Conversely, the high-intensity scenario was informed by the New York City green roof property tax abatement program, advocating an 80% coverage.

The TES determined requisite tc for achieving equity in tree planting in each Houston census tract, factoring in variables such as current tc, population, UHI severity, and demographics (e.g. income and race). In the urban tree experiments, low- and high-scenario tc was defined as 50 and 100% of the TES targeted amount. The low-scenario tc (50%) could denote either mild tree planting or a 50% tree mortality rate of high-scenario, attainable 14–17 years after planting (69). Table S3 offers a summary of the modified urban parameters, and Fig. 1 provides a schematic diagram for each experiment.

Given the pivotal role of a region's urban morphology, including factors such as roof fraction (roof area allocation for cool and green roofs) and street fraction (spaces suitable for tc), we harnessed the University of Texas-GLObal Building heights for Urban Studies (UT-GLOBUS) dataset (40). This dataset provides grid-by-grid morphological information (including building height, roof fraction, and street fraction) to the BEP–BEM, enabling more accurate urban morphology estimation for Houston.

### Validation of the UT-GLOBUS simulation setup

We conducted a thorough validation of UT-GLOBUS setup (LCZ_GLO) in comparison with the LCZ setup with default parameters (LCZ_D), leveraging MODIS land surface temperature (LST) (Fig. S3) and ISD station 2-m temperature and 2-m relative humidity (Fig. S4). Table S4 offers a summary of the default morphological parameters. Our evaluation focused on five composite heatwave events, utilizing MOD21A1 and MYD21A1 MODIS LST products with time frames featuring an 80% cloud-free grid over the urban area.

In terms of daytime LST, LCZ_GLO exhibited a higher root-mean-square error (RMSE) of 6.8 K and a mean bias (MB) of −5.0 K, in contrast to LCZ_D (RMSE: 5.9 K, MB: −4.1 K). However, for nighttime LST, LCZ_GLO displayed a higher RMSE (3.2 K) and MB (0.5 K) when compared with LCZ_D (RMSE: 3.1 K, MB: 0.8 K). Despite the greater RMSE and cooler bias of LCZ_GLO for daytime temperature, it managed to capture the spatial pattern of temperature variability more effectively (Fig. S3c and f) for both daytime and nighttime LST. Notable temperature peaks were observed in the street at the center of Houston, southwest, and north of Memorial Park (Fig. S3a and d). In contrast, LCZ_D (Fig.S3b and e) only exhibited elevated temperatures along the street, particularly in northern Houston, diverging from MODIS observations. Hence, the UT-GLOBUS setup emerged as a better choice for capturing spatial temperature variability compared to the default LCZ setup.

To assess the 2-m temperature and relative humidity, ISD stations were segregated into 11 urban and 17 nonurban stations (Fig. S1a). Across stations and events, both LCZ_GLO and LCZ_D setups yield an RMSE and MB of 0.4 K (6.1%) and 0.1 K (1.8%), respectively, for nonurban stations (Fig. S4a). However, LCZ_GLO exhibited better performance in urban areas, by reducing 2-m temperature RMSE and MB from 1.0 to 0.8 K and 0.7 to 0.3 K, respectively (Fig. S4b). Temporal trends further revealed that LCZ_GLO better captured the diurnal temperature maximum and minimum, diminishing MB for diurnal maximum and minimum from 0.5 to −0.3 K and 0.9 to 0.6 K, respectively. Concerning 2-m relative humidity, LCZ_GLO displayed improved accuracy in capturing nighttime peaks, with less underestimation, reducing MB from −10.0 to −8.6%. Nevertheless, daytime troughs exhibited an overestimation ranging from 0.8 to 4.3%.

In conclusion, the UT-GLOBUS setup demonstrated a reduction in RMSE for 2-m temperature and relative humidity, the ability to capture precise temperature peaks and troughs, and proficiency in simulating temperature spatial patterns. Despite its daytime temperature underestimation in LST assessments, LCZ_GLO exhibited lower errors in 2-m temperature simulations. Given that thermal comfort is primarily contingent on 2-m temperature and relative humidity rather than LST, we ascertain that LCZ_GLO represents a better simulation setup.

### Calculation of VDCH

As recommended by the UTCI community, a UTCI of 26°C serves as the threshold indicating the onset of heat stress in the human body. We have also tested the threshold of 32°C and have found that the results show the same order of cooling efficiency among the experiments. Therefore, we define the daily cumulative heat stress as the sum of UTCI magnitudes exceeding 26°C in each hour. The equations are as follows:


(7) $$\mathrm{Heat}\;\mathrm{stress}\;(\mathrm{HS}):\;HS_{t}=\{\begin{array}{cc} \mathrm{UTCI}-26, & \mathrm{UTCI}\geq 26\\ 0, & \mathrm{UTCI}<26 \end{array}.$$

(8) $$\mathrm{Daily}\;\mathrm{cumulative}\;\mathrm{heat}\;\mathrm{stress}\;(\mathrm{DCH}):\;\mathrm{DCH}=\overset{t=24}{\underset{t=0}{\sum }}\;HS_{t},$$

where *t* represents the local time of the day. Subsequently, we compute the average of DCH (ADCH) from all simulated days (n) and heatwave events (m) for each experiment (i).


(9) $$\mathrm{Averaged}\;\mathrm{DCH}\;\mathrm{for}\;\mathrm{experiment}\;i\;(\mathrm{ADC}H_{i}):\;\mathrm{ADC}H_{i}={\left(\frac{1}{\mathrm{nevents}\cdot \mathrm{ndays}}\overset{m=\mathrm{nevents}}{\underset{m=0}{\sum }}\;\overset{n=\mathrm{ndays}}{\underset{n=0}{\sum }}\;\mathrm{DCH}\right)}_{i}.$$

Finally, we determine the Vulnerability-Weighted Daily Cumulative Heat Stress (VDCH) for each strategy to facilitate a comparison of heat stress mitigation while considering community resilience. This is achieved by multiplying the percentage change of ADCH for each mitigation strategy (i) in relation to the CTL experiment with the SVI through a weighting (w). In this study, we use *w* = 1 to weigh the contribution from SVI and ADCH equally. However, the weighting can be modified based on the priority of vulnerability that would like to be considered. The SVI data in the census tract resolution were rasterized and regrided at 1 km resolution, matching with the spatial resolution of the innermost domain in the WRF simulation (d03) for performing the grid-by-grid calculations of VDCH. The SVI was created by the US Centers for Disease Control and Prevention, which considered four theme variables: (i) socioeconomic status (e.g. poverty and unemployment), (ii) household characteristics (e.g. elders and children), (iii) racial and ethnic minority status (e.g. Hispano and Asian), and (iv) housing type and transportation (e.g. multiunit structures and mobile homes). All SVI theme variables were weighted the same and ranked among census tracts within each state. For the details of the methodology of SVI creation, refer to the SVI documentation (21).


(10) $$\begin{array}{rl} & \mathrm{Vulnerability}\text{-}\mathrm{weighted}\;\mathrm{daily}\;\mathrm{cumulative}\;\mathrm{heat}\;\mathrm{stress}\;\mathrm{change}\;\\ & (\mathrm{VDC}H_{i}):\;\mathrm{VDC}H_{i}=w\cdot \mathrm{SVI}\times \frac{\mathrm{ADC}H_{i}-\mathrm{ADC}H_{\mathrm{CTL}}}{\mathrm{ADC}H_{\mathrm{CTL}}}\times 100\text{\%}. \end{array}$$

For instance, when considering a VDCH of −1% with SVI = 1, a 1% reduction of ADCH was achieved through this strategy. However, in neighborhoods with an SVI = 0.1, to achieve an equivalent VDCH reduction, a 10% reduction in ADCH is required due to their lower vulnerability. This can penalize the cooling impacts in less vulnerable neighborhoods because cooling in these neighborhoods brings less benefits.

## Supplementary Material

pgae360_Supplementary_Data

## Data Availability

All data generated in this study as well as a minimal dataset have been deposited in the Zenodo database https://doi.org/10.5281/zenodo.8349161. The codes for generating the plots in this study are available at: https://github.com/samuelkyfung/HeatEquity/.
