# Supplementary material for: Prioritizing social vulnerability in urban heat mitigation
Source: PNAS Nexus. 2024 Aug 30;3(9):pgae360. doi: 10.1093/pnasnexus/pgae360 (PMC11388001; doi:10.1093/pnasnexus/pgae360)
Supplement: pgae360_Supplementary_Data [file pgae360_supplementary_data.docx]

Prioritizing social vulnerability in urban heat mitigation

Kwun Yip Fung^1,2,3^, Zong-Liang Yang^1,*^, Alberto Martilli^4^, E. Scott Krayenhoff^5^, Dev Niyogi^1,6^

^1^ Department of Earth and Planetary Sciences, Jackson School of Geosciences, The University of Texas at Austin, Austin, Texas, USA

^2^ Now at: Cooperative Institute for Marine and Atmospheric Studies, University of Miami, USA

^3^ Now at: Hurricane Research Division, Atlantic Oceanographic and Meteorological Laboratory, NOAA, USA

^4^ Atmospheric Modelling Unit, Environmental Department, CIEMAT, Madrid, Spain

^5^ School of Environmental Sciences, University of Guelph, Guelph, ON N1G 2W1, Canada

^6^ Department of Civil, Architectural, and Environmental Engineering, Cockrell School of Engineering, The University of Texas at Austin, Austin, Texas, USA

*Paste corresponding author name here

Zong-Liang Yang

**Email:**  liang@jsg.utexas.edu

**This PDF file includes:**

Figures S1 to S4

Tables S1 to S4


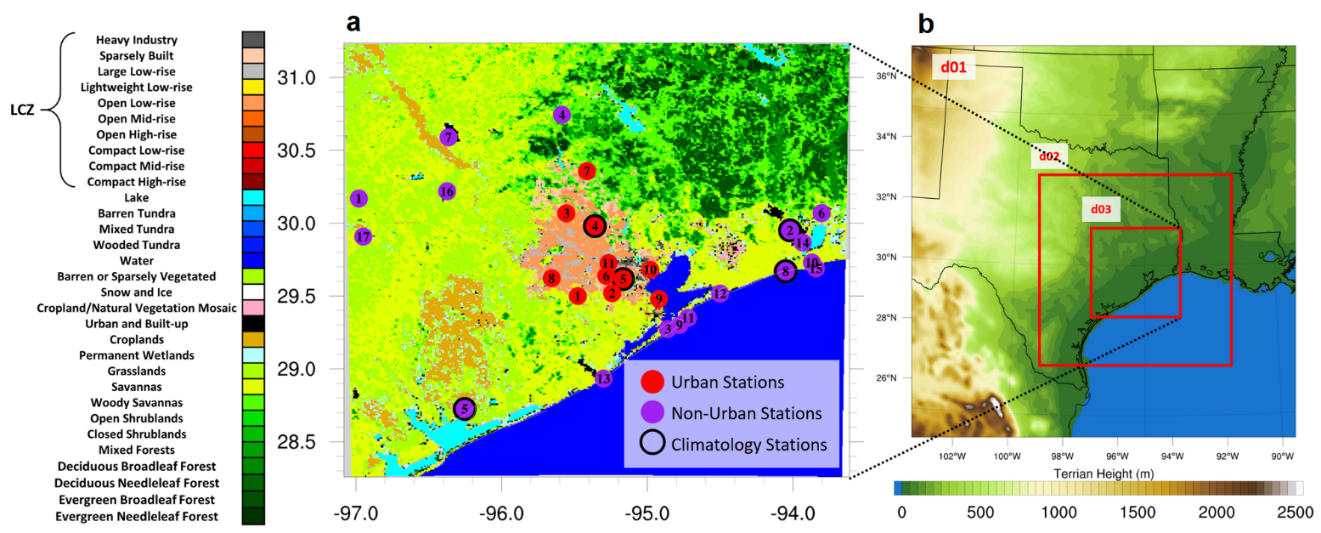


Fig. S1. Model setup. a The land use land cover map of the innermost domain. Red and purple circles indicate the urban and non-urban ISD stations, respectively. The black halo indicates the climatology ISD stations with more than 30 years of continuous data. b Model nested domains and terrain topography.


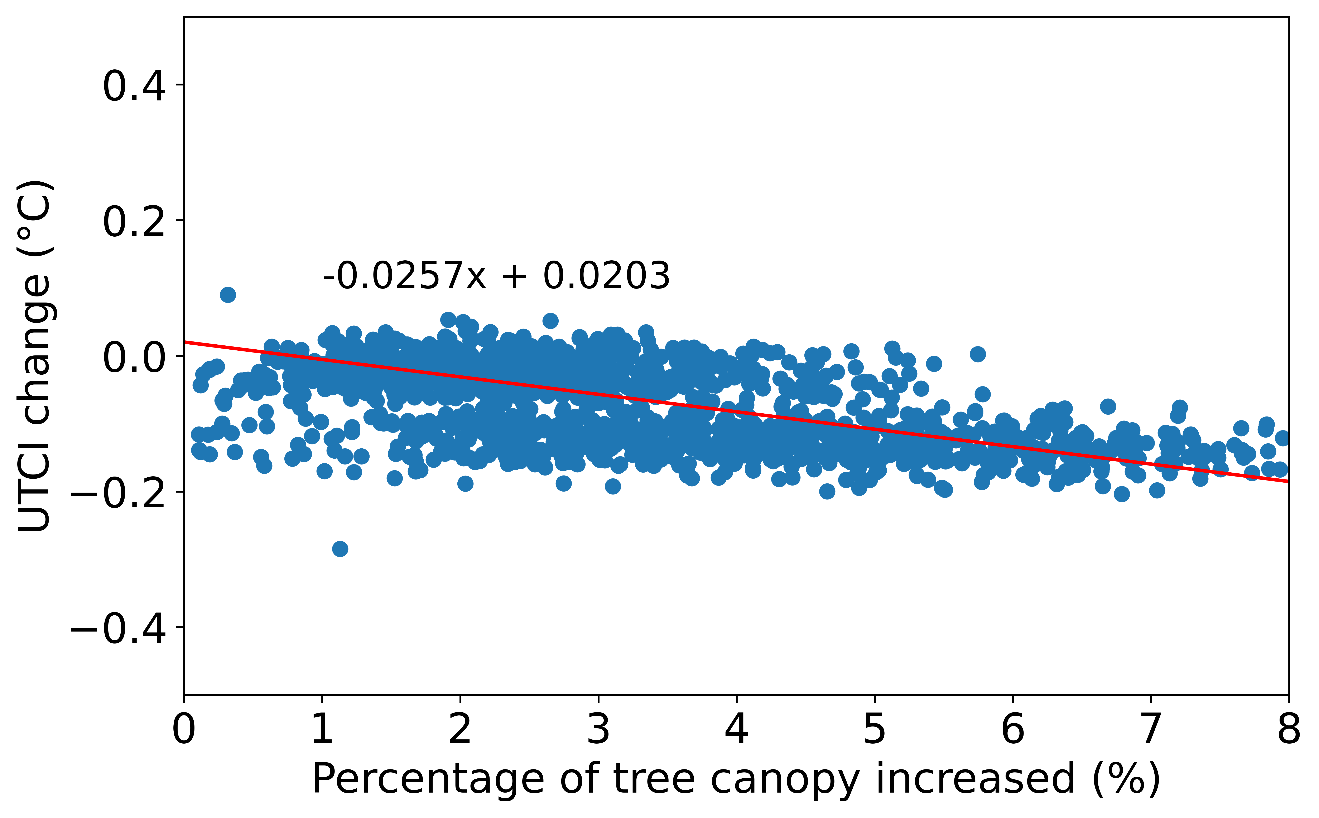


Fig. S2. The UTCI changes against tree canopy increased for urban trees experiments – control experiment. Each data point represents the case-averaged time-averaged UTCI changes in each urban grid point.


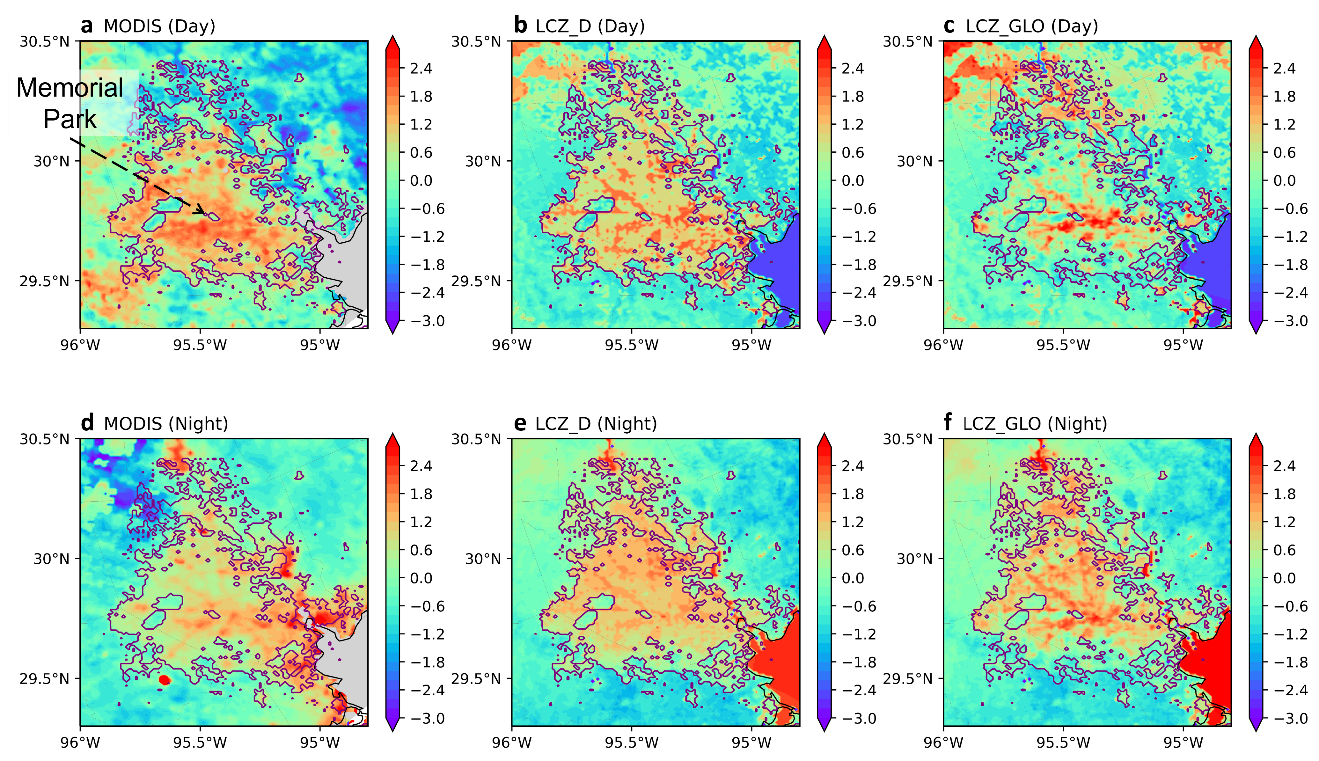


Fig. S3. The case composite of land surface temperature. a MODIS observation in the daytime. b Simulation results from LCZ with default parameters simulations in the daytime. c Simulation results from LCZ with UT-GLOBUS in the daytime. d MODIS observation in the nighttime. e Simulation results from LCZ with default parameters simulations in the nighttime. f Simulation results from LCZ with UT-GLOBUS in the nighttime.


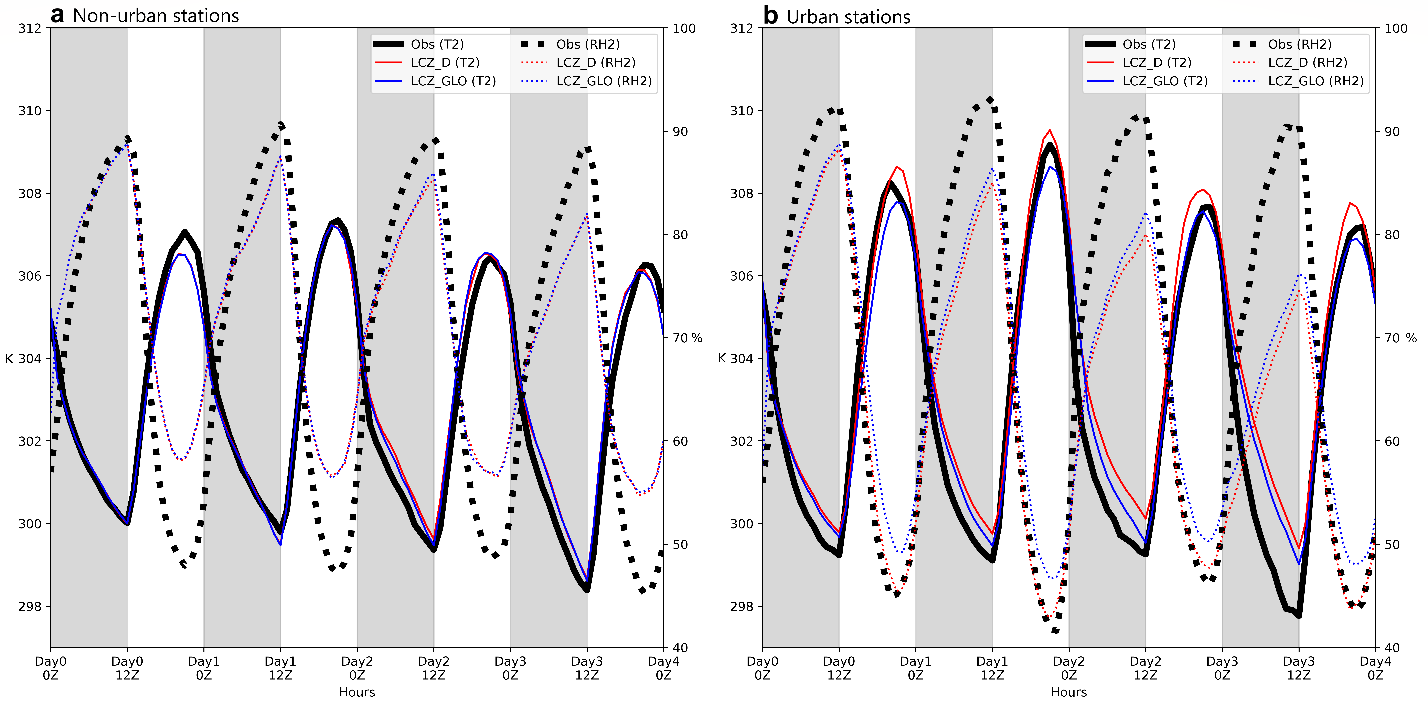


Fig. S4. The case and station composite of 2-m temperature and 2-m relative humidity. a Results from non-urban stations. b Results from urban stations. The solid lines represent the 2-m temperature and the dotted lines represent the 2-m relative humidity.

Table S1. Information for the simulated five heatwave events.

| **Events** | | | ***HW1*** | ***HW2*** | ***HW3*** | ***HW4*** | ***HW5*** |
| --- | --- | --- | --- | --- | --- | --- | --- |
| **Stations measurement** | *Heatwave  Start Date* | | 2017-07-19 | 2018-07-14 | 2018-08-13 | 2019-08-05 | 2019-08-29 |
|  | *Heatwave  End Date* | | 2017-08-02 | 2018-07-31 | 2018-08-29 | 2019-08-20 | 2019-09-16 |
|  | *Days* | | 14 | 17 | 16 | 15 | 18 |
|  | *Avg. Standard Deviation  (^o^C)* | | 2.9 | 2.8 | 2.6 | 2.7 | 3.1 |
|  | *Avg. Daily Mean (^o^C)* | | 29.5 | 29.3 | 29.5 | 30.2 | 29.1 |
|  | *Avg. Range  (^o^C)* | | 13.1 | 12.8 | 12.1 | 11.3 | 12.3 |
|  | *Avg. Daily Max. (^o^C)* | | 33.1 | 33.8 | 33.4 | 34.2 | 33.7 |
|  | *Peak Daily Max* | *Temp (^o^C)* | 35.2 | 35.6 | 35.2 | 35.5 | 35.1 |
|  |  | *Date* | 2017-07-29 | 2018-07-23 | 2018-08-21 | 2019-08-14 | 2019-09-05 |
| **Simulations** | ***Start Time*** | | **2017-07-28,  0Z** | **2018-07-22,  0Z** | **2018-08-20,  0Z** | **2019-08-13,  0Z** | **2019-09-04,  0Z** |
|  | ***End Time*** | | **2017-08-01,  0Z** | **2018-07-26,  0Z** | **2018-08-24,  0Z** | **2019-08-17,  0Z** | **2019-09-08,  0Z** |

Table S2. Model physics used in the simulation.

|  | d01 | d02 | d03 |
| --- | --- | --- | --- |
| Grid spacing (km) | 9 | 3 | 1 |
| Radiation scheme | RRTMG | | |
| Cumulus parameterization | Tiedtke | | / |
| Microphysics scheme | New Thompson | | |
| Planetary boundary layer | BouLac | | |
| Surface layer scheme | Eta similarity | | |

Table S3. Urban canopy parameters used in the experiments.

| **Parameters common for all experiments** | | | | | | | | | |
| --- | --- | --- | --- | --- | --- | --- | --- | --- | --- |
| ***Parameters*** | | ***LCZ1*** | ***LCZ2*** | ***LCZ4*** | ***LCZ5*** | ***LCZ6*** | ***LCZ8*** | ***LCZ9*** | ***LCZ10*** |
| Road Albedo | | 0.12 | 0.13 | 0.12 | 0.13 | 0.10 | 0.13 | 0.07 | 0.13 |
| Tree Height (m) | | 7.6 | 7.6 | 8.7 | 6.8 | 8.3 | 6.6 | 12.0 | 8.0 |
| **Parameters different for different experiments** | | | | | | | | | |
| ***Experiments*** | ***Parameters*** | ***LCZ1*** | ***LCZ2*** | ***LCZ4*** | ***LCZ5*** | ***LCZ6*** | ***LCZ8*** | ***LCZ9*** | ***LCZ10*** |
| *Control (CTL)* | Roof Albedo | 0.17 | 0.16 | 0.16 | 0.18 | 0.08 | 0.19 | 0.08 | 0.20 |
|  | Green Roof Coverage (%) | 0.00 | | | | | | | |
|  | Tree Coverage (%) | 10 | 20 | 14 | 13 | 22 | 16 | 30 | 14 |
| *Cool Roofs (Low)* | Roof Albedo | 0.55 | | | | | | | |
| *Cool Roofs (High)* | Roof Albedo | 0.70 | | | | | | | |
| *Green Roofs (Low)* | Green Roof (%) | 30 | | | | | | | |
| *Green Roofs (High)* | Green Roof (%) | 80 | | | | | | | |
| *Urban Trees (Low)* | Tree Coverage (%) | 18 | 22 | 21 | 21 | 28 | 24 | 36 | 23 |
| *Urban Trees (High)* | Tree Coverage (%) | 26 | 25 | 29 | 29 | 34 | 31 | 42 | 33 |

Table S4. The default urban morphological parameters in LCZ_D.

|  | ***LCZ1*** | ***LCZ2*** | ***LCZ4*** | ***LCZ5*** | ***LCZ6*** | ***LCZ8*** | ***LCZ9*** | ***LCZ10*** |
| --- | --- | --- | --- | --- | --- | --- | --- | --- |
| **Building height (m)** | 50.0 | 17.5 | 20.0 | 17.5 | 6.4 | 6.4 | 6.4 | 10.0 |
| **Building width (m)** | 22.2 | 22.0 | 42.9 | 26.3 | 13.0 | 28.9 | 43.3 | 23.8 |
| **Street width (m)** | 20.0 | 14.0 | 50.0 | 35.0 | 13.0 | 32.5 | 43.3 | 28.6 |
